# The Antibacterial Activity of Acetic Acid against Biofilm-Producing Pathogens of Relevance to Burns Patients

**DOI:** 10.1371/journal.pone.0136190

**Published:** 2015-09-09

**Authors:** Fenella D. Halstead, Maryam Rauf, Naiem S. Moiemen, Amy Bamford, Christopher M. Wearn, Adam P. Fraise, Peter A. Lund, Beryl A. Oppenheim, Mark A. Webber

**Affiliations:** 1 Queen Elizabeth Hospital, University Hospitals Birmingham NHS Foundation Trust, Birmingham, United Kingdom; 2 NIHR Surgical Reconstruction and Microbiology Research Centre, Queen Elizabeth Hospital, Birmingham, United Kingdom; 3 Institute of Microbiology and Infection, School of Biosciences, College of Medical and Dental Sciences, University of Birmingham, Edgbaston, Birmingham, United Kingdom; 4 Birmingham Children’s Hospital, Birmingham, United Kingdom; 5 The Healing Foundation Burns Research Centre, Birmingham, United Kingdom; University Roma Tre, ITALY

## Abstract

**Introduction:**

Localised infections, and burn wound sepsis are key concerns in the treatment of burns patients, and prevention of colonisation largely relies on biocides. Acetic acid has been shown to have good antibacterial activity against various planktonic organisms, however data is limited on efficacy, and few studies have been performed on biofilms.

**Objectives:**

We sought to investigate the antibacterial activity of acetic acid against important burn wound colonising organisms growing planktonically and as biofilms.

**Methods:**

Laboratory experiments were performed to test the ability of acetic acid to inhibit growth of pathogens, inhibit the formation of biofilms, and eradicate pre-formed biofilms.

**Results:**

Twenty-nine isolates of common wound-infecting pathogens were tested. Acetic acid was antibacterial against planktonic growth, with an minimum inhibitory concentration of 0.16–0.31% for all isolates, and was also able to prevent formation of biofilms (at 0.31%). Eradication of mature biofilms was observed for all isolates after three hours of exposure.

**Conclusions:**

This study provides evidence that acetic acid can inhibit growth of key burn wound pathogens when used at very dilute concentrations. Owing to current concerns of the reducing efficacy of systemic antibiotics, this novel biocide application offers great promise as a cheap and effective measure to treat infections in burns patients.

## Introduction

Local infections and burn wound sepsis are key concerns in the treatment of thermally injured patients [1], and are closely correlated to mortality and morbidity [2,3]. Despite careful treatment and infection control practices in such patients, burn wounds are readily colonised with a range of pathogenic micro-organisms, vastly increasing risks of systemic infection and graft failure [1]. Consequently, systemic sepsis resulting from invasive infection remains the leading cause of death among those with burn wounds.

Whilst the aetiology of burn wound colonisation and infection varies between specialist burn units, the most frequently implicated bacteria are *Pseudomonas aeruginosa*, *Acinetobacter baumannii*, *Staphylococcus aureus*, *Enterococcus faecalis*, *Escherichia coli*, *Klebsiella pneumoniae*, and *Enterobacter* spp [4, 5]. Of these, *P*. *aeruginosa* and *A*. *baumannii* are most prevalent [2], with Lawrence [6] finding *P*. *aeruginosa* in one-third of burn wounds, and in 59% of those patients with extensive burns. Yali *et al* [7] took clinical samples from burns patients in burn intensive care units and common burn wards and identified 1621 pathogens from 2395 clinical samples. In the burn intensive care unit, 74.2% of the pathogens were Gram-negative, and *A*. *baumannii* and *P*. *aeruginosa* were the most prevalent, representing 34.4%, and 17.7% of all the organisms in this setting, respectively. *P*. *aeruginosa* was most prevalent in the common burn wards at 12.3%.

*P*. *aeruginosa* is ubiquitous in the environment and may enter a clinical setting via the domestic water supply, *A*. *baumannii* is a particular problem in hospitals and can also persist in the hospital environment for extended periods. Dressings, topical antibacterial agents and antibiotics are used to prevent or reduce the chances of wound colonisation as well as treating infections.

Owing to the reducing efficacy of antibiotics against a large number of nosocomial pathogens (due to the emergence of resistant organisms) [8], the high doses required to treat organisms growing in sessile biofilms, and the realisation that routine use of antibiotics does not prevent bacterial colonisation [9], there is much interest in the use of novel biocide applications to prevent or reduce microbial contamination and bacterial loads in burns and wounds. A range of biocides have been investigated in this regard (e.g. silver nitrate, mafenide acetate, povidine iodine, silver sulfadiazine and chlorhexidine), including acetic acid (CH_3_COOH).

Acetic acid (AA), or vinegar, has been used sporadically in medicine for the past 6000 years [1,10], being successfully implemented to treat plague, ear, chest, and urinary tract infections [11,12,13], and in the elimination of *Bacillus pyocyaneus* (now *Pseudomonas aeruginosa*) from war wounds [2]. Furthermore, there are reports of use as a general disinfectant [14], and in the elimination of bacteria from fresh produce [15].

AA has been used for a decade in our burns centre at a concentration of 2.5% to treat patients with burn wounds infected or heavily colonised with *P*. *aeruginosa*. Here it is applied topically within dressings, is well-tolerated by patients, and is observed to have good clinical outcomes. Additionally, AA is currently used in a number of lesser economically developed countries (LEDCs) and other resource-limited settings for burn wound management.

Although there is a scarcity of literature, several small-scale clinical trials have been performed which have shown effectiveness of AA against wound infection [1,10,16,17]. In one study by Sloss *et al* [16], 16 patients with *P*. *aeruginosa-* infected burns or ulcers were recruited and treated with sterile gauze soaked in 1–5% AA, applied for 15 minutes twice daily, for 14 days. Over the study period, swabs were taken to assess the elimination of organisms from the wounds, and tests performed to assess the minimum inhibitory concentration (MIC) of AA needed to inhibit the growth of each *P*. *aeruginosa* isolate. Of the 16 patients, *P*. *aeruginosa* was eliminated from ten (63%) within seven days and from five more (31%) within 14 days. There was one eradication failure, where the burn wounds of one patient remained colonised with *P*. *aeruginosa* despite 28 days of treatment, and the authors also observed that wounds remained colonised with *S*. *aureus* and *Proteus* spp despite AA treatment.

A further study by Ryssel *et al*. [1] assessed the activity of 3% AA (selected since concentrations higher than this have been associated with severe pain and itching [9,16]) against a range of Gram-negative and Gram-positive bacterial strains isolated from patients in their burn unit. Overnight cultures of the organisms were exposed to 3% AA for 5, 30, and 60 minutes at 37°C before being diluted and covered with agar. After 48 hours incubation, the numbers of colony-forming units were counted, and analysis revealed good activity of AA, with the majority of the organisms (*Proteus vulgaris*, *P*. *aeruginosa*, *A*. *baumannii*, ß-haemolytic *Streptococci* A and B, *S*. *epidermidis*, *S*. *aureus*, and *Enterococcus faecalis)* eradicated after just 30 minutes of exposure. Additionally, Cortesia *et al* [18] have shown AA to be an effective tuberculocidal disinfectant, with 30 minutes of exposure to 6% AA resulting in an 8-log_10_ reduction in viable *Mycobacterium tuberculosis*.

A recent paper by Bjarnsholt *et al* [19] tested the *in vitro* ability of 0.5% or 1% AA to eradicate pre-existing biofilms of *P*. *aeruginosa* or *S*. *aureus* during 24 hour exposures. They found that *P*. *aeruginosa* biofilms were completely eradicated by the 0.5% AA, but that *S*. *aureus* required the higher dose of 1% AA for complete eradication.

Although these clinical studies and our observations provide evidence in support of the clinical utility of AA, the small sample sizes and heterogeneous nature of the study designs make it difficult to draw conclusions and furthermore there is little data about the antibacterial nature of AA. Fraise *et al* [20] performed the first *in vitro* assessments of the antibacterial activity and stability of AA, where they determined the MIC of AA against a range of organisms, as well as any potential inhibition of action due to evaporation, the presence of cotton gauze and organic matter. They concluded that AA was effective at a far lower concentration than initially indicated by previous studies (with MICs of 0.16% against *P*. *aeruginosa*), and that there was no reduction in activity due to evaporation and cotton gauze/organic matter.

Despite Fraise *et al* [20] providing the first robust experimental data of the ability of AA to prevent the growth of a range of important nosocomial pathogens, a key limitation was the focus on planktonic growth. Since it is estimated that 80% of all infections include at some point the infective bacteria existing as a biofilm [21], and bacteria in a biofilm are significantly more antibiotic and biocide resistant than planktonic cells [21], the current study was undertaken to further investigate the *in vitro* antibacterial activity of AA against important burn wound colonising organisms growing planktonically and as sessile biofilms.

We assessed the ability of AA to both prevent formation of a biofilm (to reduce colonisation of a wound), and to eradicate pre-formed biofilms (to treat colonised wounds).

## Methods

A series of *in vitro* experiments were conducted with a panel of organisms (Table 1) to determine the efficacy of acetic acid against planktonic (free-floating in broth) and biofilm (attached to a surface) growth. The strains comprised well-characterised control strains and clinical isolates (including those from burn patients), and were selected because of their relevance to infection in the burn unit setting (*P*. *aeruginosa* and *A*. *baumannii*). Control strains were chosen to represent major globally relevant clonal complexes of the two species (ensuring the results are likely to be generally applied to each species as a whole). All *P*. *aeruginosa* and *A*. *baumannii* isolates were genotyped prior to the study through variable number tandem repeat analysis (VNTR) and pulsed-field gel electrophoresis (PFGE), respectively by the relevant UK reference laboratories of Public Health England.

**Table 1:** List of the control and clinical isolates used in this study.

Study Identifier	Organism	Description (genotyping results^£^)
PS_PA01	*Pseudomonas aeruginosa*	ATCC_15692 (10,2,2/4,2/5,5,3,-,3,8)
PS_6749	*Pseudomonas aeruginosa*	NCTC_6749 (11,3,2,5,4,-,4,2,10)
PS_27853	*Pseudomonas aeruginosa*	ATCC_27853 (11,4,5,2,3,2,-,4,7)
PS_919	*Pseudomonas aeruginosa*	QEHB Clinical burn isolate (10,2,5,4,6,2,-,4,-)
PS_927	*Pseudomonas aeruginosa*	QEHB Clinical burn isolate (10,2,5,4,6,2,10,4,12)
PS_1054	*Pseudomonas aeruginosa*	QEHB Clinical burn isolate (11,6,2,2,1,3,7,2,11)
PS_1586	*Pseudomonas aeruginosa*	QEHB Clinical burn isolate (11,4,5,2,2,3,8,2,13)
PS_1587	*Pseudomonas aeruginosa*	QEHB Clinical burn isolate (10,3,5,5,4,1,3,7,7)
AB_19606	*Acinetobacter baumannii*	ATCC_19606 (unique)
AB_17978	*Acinetobacter baumannii*	ATCC_17978 (unique)
AB_1a	*Acinetobacter baumannii*	QEHB Clinical burn isolate (QUEE13AC-27)
AB_53	*Acinetobacter baumannii*	QEHB Clinical burn isolate (QUEE13AC-27)
AB_AYE	*Acinetobacter baumannii*	MPR Clinical Isolate (unique)
AB_C58	*Acinetobacter baumannii*	NCTC_13305 (unique)
AB_C59	*Acinetobacter baumannii*	NCTC_13420 (S E Clone)
AB_C60	*Acinetobacter baumannii*	NCTC_13424 (unique)
EC_073	*Escherichia coli*	EPEC CFT_073
EC_042	*Escherichia coli*	EAEC_042
PM_421	*Proteus mirabilis*	QEHB Clinical wound isolate
MSSA_10788	*Staphylococcus aureus*	NCTC_10788
MRSA_12493	*Staphylococcus aureus*	NCTC_12493
MRSA_F475	*Staphylococcus aureus*	EMRSA_16
MRSA_F473	*Staphylococcus aureus*	EMRSA_15
MDR_A	CPE^ *Klebsiella pneumoniae* (NDM-1^+^ positive)	QEHB Clinical isolate
MDR_B	CRE* *Klebsiella pneumoniae* (ESBL positive with additional permeability changes)	QEHB Clinical isolate
MDR_C	*E*. *coli* (ESBL^@^ positive)	NCTC_13451
MDR_D	*Pseudomonas aeruginosa* (VIM^$^ positive)	Royal Free Hospital Clinical isolate
MDR_E	CRE *Enterobacter cloacae* (AmpC^#^ positive with additional permeability changes)	QEHB Clinical isolate
MDR_F	CRE *Enterobacter cloacae* (AmpC positive with additional permeability changes)	QEHB Clinical isolate

^ Carbapenemase producing Enterobacteriaceae

^+^ New Delhi metallo-β-lactamase

* Carbapenem resistant Enterobacteriaceae

^@^ Extended-Spectrum β-lactamase

^$^ Verona integron-encoded metallo-β-lactamase

^#^ AmpC-type β-lactamase

^£^ genotyping refers to VNTR profile for *P*. *aeruginosa* and PFGE data for *Acinetobacter*.

For *A*. *baumannii* we included representatives of seven distinct strains, including two isolates of profile QUEE13AC-27 (previously found in the hospital), and one isolate that clusters with the South East clone (a sub-lineage of International Clone II that has been prevalent in the past). Strain AYE was also included as a representative of International Clone I, the other major globally relevant lineage (Table 1). The *P*. *aeruginosa* isolates represent seven distinct strains. Of note, PS_1054 and PS_1587 are representatives of clones C and D, both of which are frequently isolated in the UK [22]. We aimed to test diverse isolates in preference to large numbers of related strains, and recent isolates from burns patients were included to ensure no differences were seen in isolates from typical patient specimens. We also included a range of other 'comparator' organisms commonly causing hospital acquired infection including *E*. *coli*, *P*. *mirabilis*, *S*. *aureus*, *K*. *pneumoniae* and *Enterobacter cloacae*. The panel also included control strains of *S*. *aureus* (NCTC 10788, NCTC 12493) and *P*. *aeruginosa* (NCTC 6749, ATCC 27853), since these are recognised test strains in the EN standards for assessing the efficacy of chemical disinfectants (e.g. EN 13727 [23]). All isolates were characterised (and were varied) in terms of antibiogram (data not shown), were stored at -80°C on Protect beads, and were routinely cultured on cysteine lactose electrolyte deficient (CLED) agar prior to each experiment.

Experiments were designed to assess i) the antibacterial activity of AA in terms of its minimum inhibitory concentrations (MIC) against planktonic growth, and ii) activity against biofilming organisms, both in terms of prevention of formation, and destruction of pre-formed biofilms. Acetic acid supplied at 5% w/v [Tayside Pharmaceuticals, Dundee, UK] was used as a stock for all experiments.

### Determination of the Minimum Inhibitory Concentration of acetic acid

Susceptibility to AA was assessed using fresh overnight lysogeny broth (LB) [Sigma-Aldrich, UK] cultures of the organisms, which were diluted in Iso-sensitest (ISO) broth [Oxoid, Basingstoke, UK], and seeded (50μl) into 96-well microtiter trays (MTT) at a concentration of approximately 10^5^ colony forming units/ml. Acetic acid (5% w/v) was then diluted in ISO broth to produce a range of final biocide/inocula concentrations of 2.5%,1.25%, 0.63%, 0.31%, 0.16% and 0.08%. 50μl of these concentrations were then added to the test organisms in the MTT and the volume of each well made up to 150μl through the addition of a further 50μl of ISO broth. Therefore per test well the concentrations of AA contained in the 150μl ranged from 1.6–0.026%. Since in an *in vivo* setting the amount of wound exudate would not be standard, all results were reported based on the initial inocula concentrations.

Controls were included for each assay, comprising 50μl diluted overnight cultures of the organisms, plus 100ul ISO broth (for the positive control), or 150μl ISO broth alone (for the negative control). Three technical replicates were performed for each AA concentration, and each organism tested in duplicate.

The MICs were read visually after 18 hours static incubation at 37°C. Data from MTTs where the negative control wells were turbid were rejected.

### Impact of acetic acid on biofilm formation

The concentration of AA needed to prevent biofilm formation was assessed using a crystal violet biofilm formation assay as described by Baugh *et al* [24], with the endpoint measurement being the ‘minimum biofilm inhibitory concentration’ (MBIC). Overnight LB cultures of all the test strains were diluted in fresh antibiotic-free Mueller-Hinton (MH) broth [Oxoid] to an optical density at 600nm (OD_600_) of 0.1, and then 100μl seeded into wells of a 96-well MTT, alongside 100μl of either diluted AA (water as diluent) or sterile water. AA was tested at the following inocula concentrations: 5%, 2.5%, 1.25%, 0.63%, 0.5%, 0.4%, 0.31%, 0.16%, 0.1%, 0.09%, 0.08%, 0.07%, 0.06%, 0.05%, 0.04%, 0.03%, 0.02% and 0.01%. This range was chosen to reflect the concentration used in clinical practice and below. Whilst the MIC is known to be at the lower end of the range we aimed to determine activity against biofilms (where susceptibility may be less), so used a range starting at 2 X that in current clinical practice (i.e. 5%). The range also included sub-inhibitory concentrations to allow assessment of any impacts against biofilm formation which may not relate to growth inhibition. Suitable controls were included in each assay, comprising 100μl overnight bacterial culture with 100μl water (for the positive control), or 200μl MH broth (for the negative control). Two biological and three technical replicates were performed for each strain and each AA dilution, respectively, and each experiment was repeated to check for reproducibility.

Plates were sealed and statically incubated at (33°C); the temperature of the surface of a wound [25]. After 72 hours, the liquid was removed from the wells and the plates rinsed in tap water to remove any unbound cells. Any existing biofilms were then visualised through staining with 200μl of 1% crystal violet (CV) [Sigma Aldrich, Poole, UK], further rinsed (as above) to remove unbound CV, and dye solubilised by the addition of 200μl of 70% ethanol. The OD_600_ of the solubilised CV solution was then measured using a FLUOstar Optima [BMG Labtech] to assess the biomass of the biofilms.

The positive and negative controls for each test plate were examined and if within a normal range the rest of the data analysed for statistical significance by comparing values at each concentration of AA to untreated (positive) controls using the students’ ‘t’ test.

### Impact of acetic acid on biofilm viability

The antibacterial activity of AA against pre-formed biofilms was also assessed by conducting ‘minimum biofilm eradication concentration’ (MBEC) experiments (as in Ceri *et al* [26]) on each isolate. Overnight LB cultures of the test strains were diluted in fresh antibiotic-free MH broth to an OD_600_ of 0.1 and then 200μl seeded into wells of a 96-well MTT. Positive (200μl 0.1 OD_600_ diluted organisms) and negative (200μl MH broth) controls were included for each MTT.

To produce a ‘transferable biofilm’, a 96 well polypropylene plate [Starlabs, UK] was then placed into the MTT so that each well contained a ‘peg’, on which biofilms could form, before the plates were sealed, and statically incubated at 33°C for 72 hours. After 72 hours, the pegs (±biofilm) were removed and washed in a MTT containing sterile water (to remove any unbound cells), and the ‘peg plate’ placed into a new MTT containing either 200μl of AA (at concentrations from 5% to 0.01% diluted in water, as for the MBIC), or 200μl broth (for the positive and negative control wells). This assembly was wrapped, and left to incubate for three hours at 33°C. The peg plate was then removed from the AA, washed as before, and carefully placed into a MTT containing 200μl sterile MH broth (herein referred to as ‘reporter broth’) for overnight incubation. After 18 hours, the OD of the reporter broth was measured to assess the viability (seeding) of the biofilms following AA exposure, and to determine the minimum concentration of AA which prevented any seeding of the reporter broth and by inference had killed some of the cells in the biofilm.

To demonstrate the presence of biofilms on the pegs, CV assays were performed on the pegs after the OD of the reporter broth had been measured. This involved placing the pegs into MTTs containing 200μl of 1% CV, followed by washing and subsequent solubilisation in 200μl of 70% ethanol. The peg biofilm biomass could then be measured using OD readings as previously and the presence of the biofilm confirmed. Two biological and three technical replicates were performed for each strain and each AA dilution, respectively.

To confirm that prevention of seeding in the biofilm eradication experiment was due to loss of viability of the cells (rather than lack of release of viable cells), a sonication experiment was performed on biofilms representing each species. This involved forming biofilms on 5 ml polystyrene tubes [Deltalab, Spain] placed inside 15ml Falcon tubes [Corning Life Sciences, Netherlands], exposing the test biofilms to 3 hours of AA (and the control biofilms to water alone), and then assessing seeding as before. All biofilms were then sonicated using a MSE SoniPrep 150 platform for 10 seconds at 2.5Mhz and 200μl of the sonicate placed into 5ml LB broth for overnight incubation at 37°C. The OD of the broth was then measured as previously. These data supported the use of the reporter broth as a good measure of biofilm viability although it is not possible to exclude the potential for small numbers of viable cells in a dormant state or encased deeply within matrix not to be identified in these type of experiments.

## Results

### Determination of the Minimum inhibitory concentration of acetic acid

A total of 29 isolates were tested (nine *P*. *aeruginosa*, eight *A*. *baumannii*, and 12 comparators (Table 1)). AA was effective at preventing planktonic growth of all organisms, with MICs from 0.16–0.31%. Nine isolates (31%) had a MIC of 0.16%, with a further 20 (69%) inhibited by 0.31% AA. Consequently, all 29 isolates (100%) had an AA MIC ≤ 0.31% (Table 2).

**Table 2:** Table showing the tests performed on the isolates and their MIC, MBIC and MBEC values of AA.

Study Identifier	Organism	Tests performed^a^ (inhibitory % of AA)								
		MIC		MBIC			MBEC			
		(0.16)	(0.31)	(≤0.1)	(0.16)	(0.31)	(≤0.1)	(0.16–0.5)	(1.25)	(2.5)
PS_PA01	*P*. *aeruginosa*	✓				✓	✓ ^b^			
PS_6749	*P*. *aeruginosa*		✓		✓		✓ ^b^			
PS_27853	*P*. *aeruginosa*		✓			✓	**Not performed**			
PS_919	*P*. *aeruginosa*	✓		✓					✓ ^b^	
PS_927	*P*. *aeruginosa*	✓				✓	✓			
PS_1054	*P*. *aeruginosa*	✓		✓				✓ ^b^		
PS_1586	*P*. *aeruginosa*		✓			✓		✓ ^b^		
PS_1587	*P*. *aeruginosa*		✓			✓			✓ ^b^	
AB_19606	*A*. *baumannii*	✓		✓					✓	
AB_17978	*A*. *baumannii*		✓	**Unreliable biofilm production**			**Not performed**			
AB_1a	*A*. *baumannii*		✓		✓			✓ ^b^		
AB_53	*A*. *baumannii*		✓	✓			✓ ^b^			
AB_AYE	*A*. *baumannii*	✓			✓			✓		
AB_C58	*A*. *baumannii*		✓	**Poor biofilm formed**			**Not performed**			
AB_C59	*A*. *baumannii*	✓			✓				✓ ^b^	
AB_C60	*A*. *baumannii*	✓			✓				✓ ^b^	
EC_073	*E*. *coli*		✓		✓			✓ ^b^		
EC_042	*E*. *coli*		✓		✓					✓
PM_421	*P*. *mirabilis*		✓			✓			✓	
MSSA_10788	*S*. *aureus*		✓			✓			✓	
MRSA_12493	*S*. *aureus*		✓	**Poor biofilm formed**			**Not performed**			
MRSA_F475	*S*. *aureus*	✓		**Poor biofilm formed**			**Not performed**			
MRSA_F473	*S*. *aureus*		✓	**Poor biofilm formed**			**Not performed**			
MDR_A	CPE *K*. *pneumoniae*		✓		✓			✓		
MDR_B	CRE *K*. *pneumoniae*		✓			✓		✓		
MDR_C	ESBL *E*. *coli*		✓	✓					✓	
MDR_D	VIM *P*. *aeruginosa*		✓		✓			✓		
MDR_E	CRE *E*. *cloacae*		✓	**Unreliable biofilm production**			**Not performed**			
MDR_F	CRE *E*. *cloacae*		✓		✓				✓	
		9	20	5	10	8	4	8	9	1

^a^ ✓ denotes that the test was performed and the result.

^b^ denotes that there was statistically significant reduction in seeding at all concentrations of AA (t-test, p<0.05)

The difference in MIC between 0.16 and 0.31% for different strains of the same species was not considered significant and was not linked to any differences in antibiogram.

### Impact of acetic acid on biofilm formation

**Fig 1:**
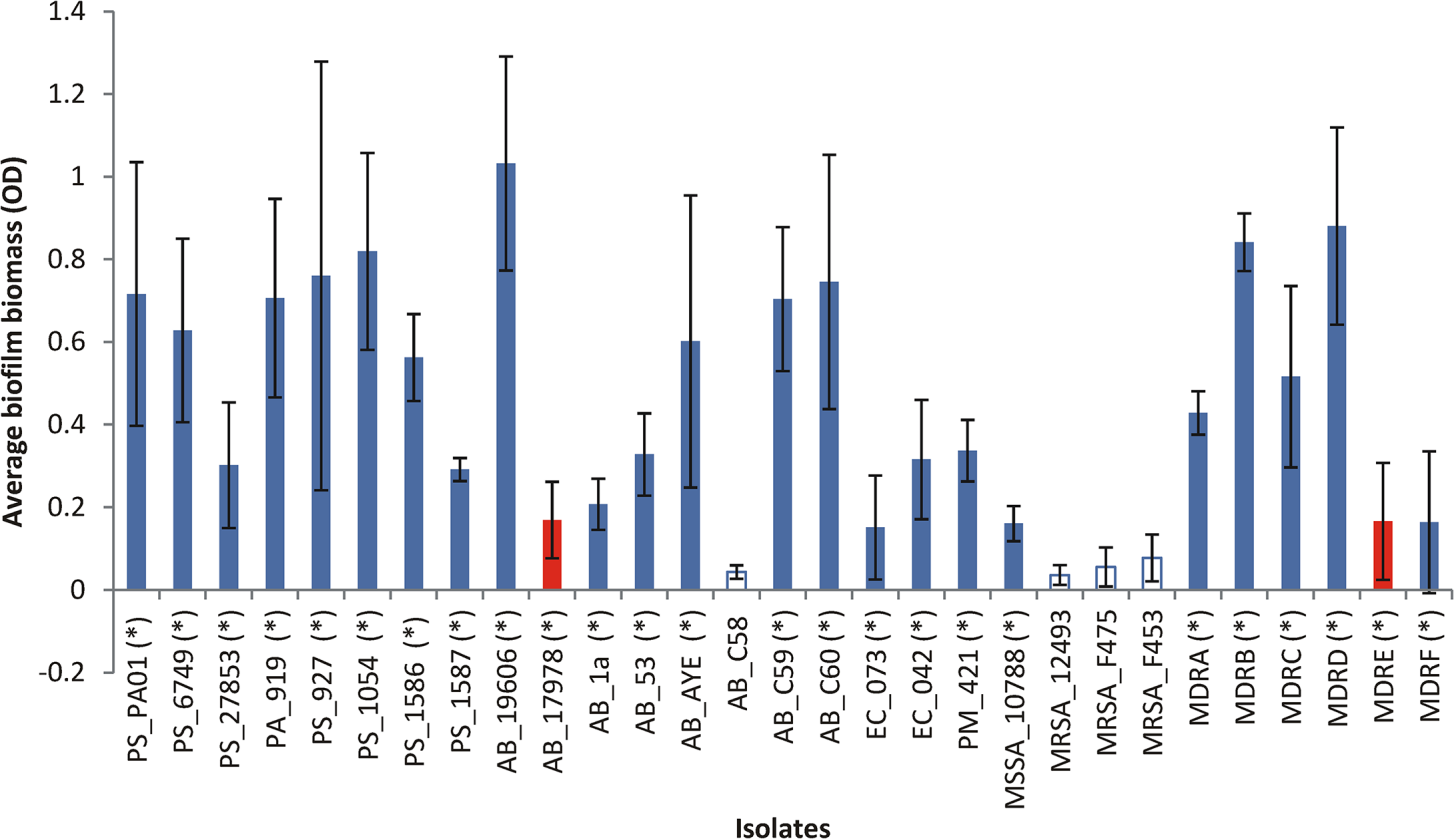
Graph showing the mean average biomass of the biofilms produced by all isolates, as measured through the crystal violet assay. Optical density on the y axis refers to the average biofilm biomass (in the absence of AA) for all the isolates shown on the x axis. White (unshaded) bars represent isolates that were excluded from further testing owing to poor biofilming ability when compared to their other species counterparts. Red bars represent the isolates with unreliable biofilm production. Error bars represent the standard error for each average value and asterisks denote values statistically significantly different from the broth only control.

The same 29 isolates were subjected to AA in the MBIC assay (Table 2). A spectrum of biofilm formation was seen, but four isolates (13.8%) (comprising *A*. *baumannii* (n = 1) and MRSA (n = 3)), were poor at producing biofilms (when compared to other isolates of the same species), and two isolates (6.9%) (comprising *A*. *baumannii* (n = 1) and MDR_E carbapenem-resistant *E*. *cloacae* (n = 1)) were deemed unreliable biofilm-producers. MDR_E was defined as being unreliable, since this isolate exhibited variable biofilm production and failed to produce a biofilm equivalent to the lowest amount produced by MSSA_10788on 25 of 44 tests. Isolates where the average biofilm formation (as measured by OD) after crystal violet staining was not significantly different (as measured by students ‘t’ test) to the broth only controls, were excluded from further analysis. This included the six isolates mentioned above (20.7%) which were not included in further analysis of anti-biofilm effects (nor tested in the MBEC model) (Fig 1).

AA was effective at preventing the formation of a biofilm for the remaining 23 isolates tested, with MBICs of i) <0.10% for five (20.8%) organisms (*P*. *aeruginosa*, A. *baumannii*, and *E*. *coli*), ii) 0.16% for 10 (43.5%) (*P*. *aeruginosa*, *A*. *baumannii*, *E*. *coli*, *K*. *pneumoniae*, and *E*. *cloacae*), and iii) 0.31% for eight (33.3%) (*P*. *aeruginosa*, *P*. *mirabilis*, and MSSA) (Table 2). Consequently 0.31% AA (or lower) was effective at preventing the formation of biofilms by all 23 isolates tested.

**Fig 2:**
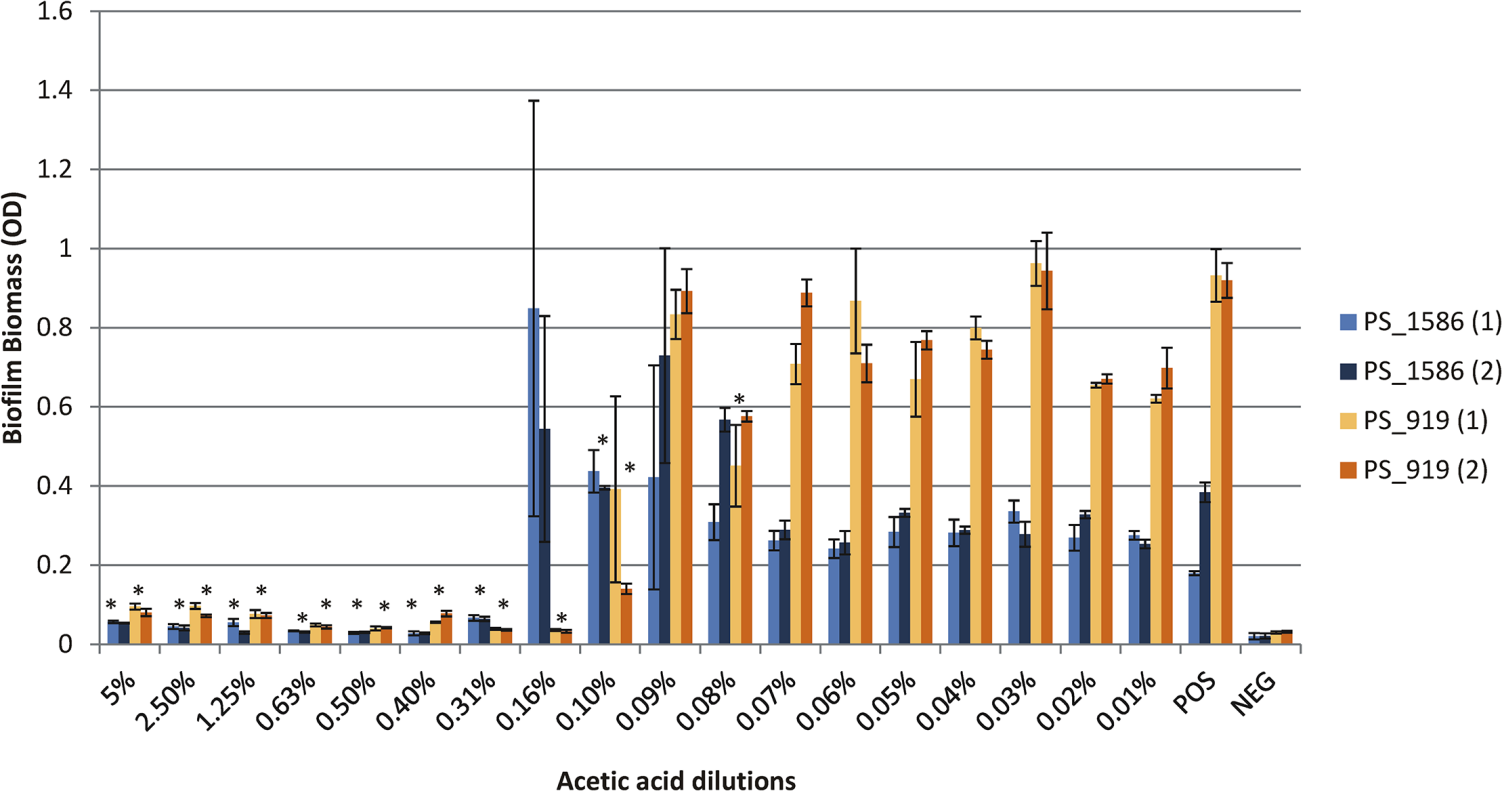
Graph showing the MBIC results for isolates PS_919 and PS_1586. Optical density on the y axis refers to the average biofilm biomass for isolates PS_919 and PS_1586 at the range of AA dilutions tested. POS: positive control, NEG: negative (broth only) control. The error bars represent the standard error, and asterisks denote dilutions with statistically significant reductions in biofilm production according to the t-test.

The degree of biofilm inhibition was dose-dependent, with the MBIC representing the lowest dilution where there is no apparent biofilm, and a statistically significant difference (p<0.05) in biofilm biomass compared to the positive control. Fig 2 shows these data for two randomly selected representative strains of *P*. *aeruginosa* (PS_919 and PS_1586). Graphs showing the MBIC for all the isolates can be found in the supplementary results (S1 Fig).

Concentrations of AA below the MIC were also analysed for biofilm formation, by measuring the Biofilm Forming Units (BFU) (A595 of the solubilized CV solution/A600 of the planktonic phase). There was no significant inhibition of biofilm formation at these low concentrations of AA (an example of these data are shown in S2 Fig).

Student t-tests performed on the data from test wells with and without AA, and at all % of AA, indicated statistically significant (p-value ≤0.05) reduction in biofilm biomass formed for i) five isolates with a MBIC of ≤0.10%, ii) 10 with an MBIC of 0.16%, and iii) eight with an MBIC of 0.31%.

### Impact of acetic acid on biofilm viability

The MBEC assay was performed on 22 biofilm-forming isolates to assess the activity of AA against pre-formed biofilms. Seven were not tested since they failed to form a biofilm in the MBIC assay (Table 2), or were identified to be a weaker biofilm-producing organism in this specific assay (PS_27853).

**Fig 3:**
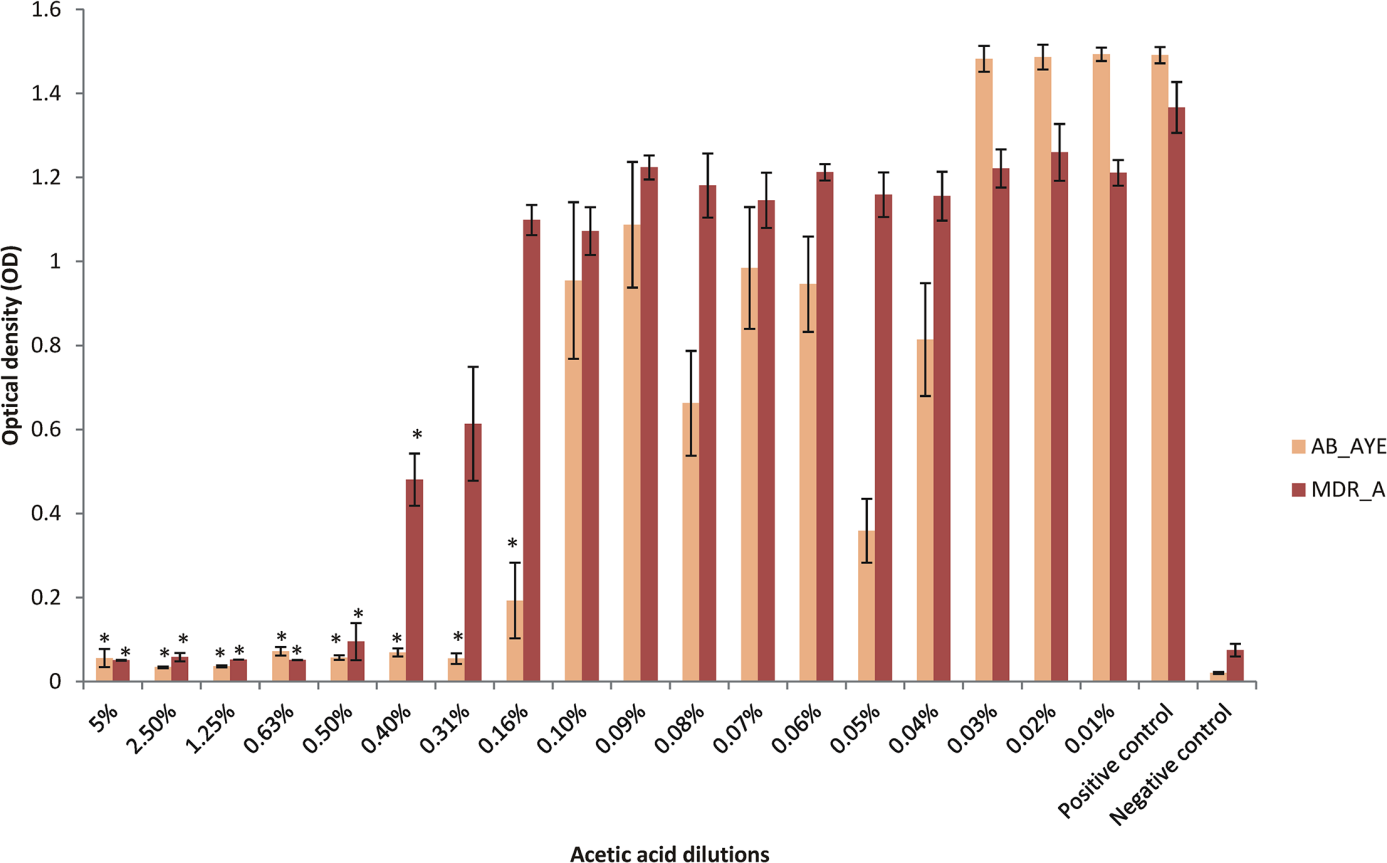
Graph showing the MBEC results for isolates AB_AYE and MDR_A. Optical density on the y axis refers to the average biofilm seeding for isolates AB_AYE and MDR_A after 3 hours of exposure to AA at the range of dilutions tested. POS: positive control, NEG: negative (broth only) control. The vertical line represents the MBEC, the error bars represent the standard error, and asterisks denote dilutions with statistically significant reductions in the seeding of the biofilm according to the t-test.

For the 22 isolates tested, incubation of the biofilm-coated peg plate for three hours at 33°C in the range of dilutions of AA (5–0.01%) resulted in a statistically significant reduction in seeding. This was visualised by plotting a graph of the detector broth OD (with high numbers indicating turbidity and hence seeding of a viable biofilm), and then measured by subjecting all AA dilutions and the positive control (the overnight bacterial cultures in the absence of AA) to the paired t-test. The lowest concentration of AA where there was minimal seeding of the biofilm and a p value of ≤0.05% was recorded as the MBEC (Table 2). Fig 3 shows randomly selected representative MBEC data for AB_AYE and MDR_A. Graphs showing the MBEC for all the isolates can be found in the supplementary results (S3 Fig).

Plots were also made of the CV values in order to prove that biofilms (viable or otherwise) were present on the pegs at the time of exposure to AA and the reporter broth. Biofilms were present on all the test pegs in the MBEC assay and hence the reduction in seeding that we observed is a true reflection of the effect of the 3 hour AA exposure.

The concentrations of AA that eradicated a pre-formed biofilm ranged from ≤0.10% to 2.5% (Table 2). In addition to this complete killing of the biofilm, there was statistically significant reduction in seeding at all dilutions of AA for 11 organisms (*P*. *aeruginosa*, *A*. *baumannii* and *E*. *coli*) (Table 2). For the others, the t-test data, and MBEC values read from the graphs, were largely in agreement (data not shown).

## Discussion

Acetic acid is used (on a case-by-case basis) in our burns unit at a concentration of 2.5% and has been anecdotally observed to reduce bacterial loads whilst being well tolerated by patients. Our *in vitro* study supports these data, with laboratory results showing that AA is capable of preventing the planktonic and biofilm growth of all twenty-nine isolates at AA dilutions of 0.31% for the MIC and MBIC, and ≤2.5% for the MBEC (with the majority of biofilms eradicated at ≤1.25%). Whilst the methodology used in our study does not investigate the physiology of every individual cell within a biofilm it is clear the AA has a major impact on both biofilm formation and viability of mature biofilms.

AA was active against the Gram-positive organisms tested to date, with MICs of 0.16% and 0.31% for the broth MIC, 0.31% for the MBIC and 1.25% for the MBEC, but generally the Gram-negative isolates were more susceptible. These included those commonly encountered (e.g. *P*. *aeruginosa* and *A*. *baumannii*), as well as the multi-drug resistant strains (e.g. those producing extended-spectrum beta-lactamases, and the carbapenemase producing Enterobacteriaceae). Interestingly, there was no difference in activity against multi-drug resistant organisms compared to non multi-drug resistant strains. With the ever-limited antibiotic choices for these organisms, AA is a promising topical therapy option, and an ideal way to conserve use of critical systemic antibiotics.

Of key interest, AA can also eradicate a pre-formed biofilm, with three hour exposures to the range of AA dilutions resulting in a statistically significant (p<0.05) reduction in seeding. The levels of AA for the MBEC were mostly higher than seen for the MBIC; this is not surprising given that we know bacteria in a biofilm are significantly more antibiotic and biocide resistant than planktonic cells [21]. For some isolates the MBEC and MBIC values were very similar and on four occasions MBEC values were lower than MBIC values although within one dilution in each case. This represents the accepted error for MIC experiments indicating it is unlikely there is a real significant difference in these values. It is however possible this represents physiological differences in the mature biofilms which may be more susceptible than actively growing cells to AA for currently unknown reasons.

Although this study has not provided any insight into the mechanism of action of AA, it is clear that AA concentrations from 5% (pH ~2.3, pKa = 4.75)) to as low as 0.31% (pH ~2.9) are sufficient to prevent both planktonic growth, and eradicate biofilms formed by a range of organisms of direct relevance to patients with burns. As expected, the effect of AA was not dependent on pH alone, with experiments performed using matched pH solutions (pH of 2.9 and 2.4) of hydrochloric (HCl) acid (inorganic) and acetic acid (organic) indicating that at the same pH, HCl was unable to prevent biofilm growth of two strains of *P*. *aeruginosa* (PS_PA01 and PS_1054) and *A*. *baumannii* (AB_AYE, AB_C59) whilst AA was effective (S4 Fig). These findings are consistent with those of Bjarnsholt *et al* [19] who took 24-hour old cultures of *P*. *aeruginosa* and treated them with 0.5% AA solutions with increasing pH (through the addition of NaOH). The range of pH of these test solutions was 4.33 to 6.0 and they found complete killing of all bacteria in the wells when the pH was lower or equal to 4.33 (above pH 5 there were only small, non-significant effects). In line with our findings, when the same experiment was performed with hydrochloric acid (in the exact same range of pH), there was no microbial killing at all (data not shown).

It has long been known that the toxicity of weak acids towards bacteria such as acetic acid to cells is not just a consequence of their acidity [27, 28, 29]. They are thought to exert their toxic effects through a variety of mechanisms. Weak acids can cross bacterial membranes more readily than strong acids, because of the equilibrium between their ionised and non-ionised forms, the latter of which can freely diffuse cross hydrophobic membranes [30, 31]. A consequence of this is that they tend to collapse the proton gradients that are necessary for ATP synthesis, as free anions (acetate in this case) will combine with periplasmic protons pumped out by the electron transport chain, and carry them back across the membrane without passage through the F1Fo ATP synthase. However, as the internal pH of the cell (typically around pH 7.6 [32, 33] in neutralophilic bacteria) is higher than that of the weak acid solution outside the cell, the internalised acetic acid will dissociate, acidifying the cytoplasm, which in turn can cause acid-induced protein unfolding, membrane, and DNA damage [28, 34]. The anion released by this process is a separate cause of toxicity, which may be caused by a variety of events, such as osmotic stress to the cell. The nature of these is known to depend on the particular anion, though the mechanistic reasons for this are not fully understood; thus, different weak acids at the same pH can have very different toxic effects on cells [27, 28, 30]. Our results and the recent results of Bjarnsholt *et al*. [19] are consistent with and indeed predicted from these previous findings.

## Conclusions

This study demonstrates that AA is a potential alternative to antibiotics and traditional antimicrobial dressings for preventing colonisation of burns, and may have a role in the management of burns in both developed and especially developing countries. Although currently the use of AA in the clinical setting is limited owing to factors including concerns of tolerability and toxicity, we have shown that AA is effective at far lower concentrations than previously reported [1, 16, 18]. It is reasonable to envisage that there will be lower toxicity with lower concentrations of AA.

Controlled clinical trials in the treatment of burn infections (especially looking at tolerability and toxicity) with AA are warranted as this promises to be a cheap and efficacious treatment option for controlling colonisation and preventing infections in patients with burn wounds. A clinical trial has been approved for this purpose at our centre and data from this study is being used to assess the concentrations of AA to test in that trial. Further experimental work should extend the panel of organisms to be tested to include Gram positive organisms such as *S*. *aureus* and *Enterococcus* spp., and to test activity of AA on mixed genera biofilms. It is also pertinent to assess efficacy, tolerability and toxicity of AA *in vivo* (when used at the lower effective concentrations reported in this paper) and investigate the likeliness of resistance developing in bacteria being exposed to AA, although this has not been seen to date in our clinical experience.

## Supporting Information

S1 Fig MBIC data for all isolates. Panels of isolates as follows; A: *P*. *aeruginosa*, B: *A*. *baumannii*, C: MDR, D: all other comparators
(TIF)

S2 Fig Broth OD (graph A), biofilm biomass (graph B) and Biofilm forming units (graph C) for four *A*. *baumannii* strains. NB: Biofilm forming units were calculated by dividing the A595 of the solubilised CV solution by A600 of the planktonic (broth) phase. Optical density on the y axis refers to the average quantity of planktonic bacteria (graph A), and the biofilm biomass (graph B) for all isolates tested at the range of AA dilutions tested. The error bars represent the standard error.
(TIF)

S3 Fig MBEC data for all isolates. Panels of isolates as follows; A: *P*. *aeruginosa*, B: *A*. *baumannii*, C: MDR, D: all other comparators.
(TIF)

S4 Fig Graph showing the MBIC results for two *P*. *aeruginosa* and two *A*. *baumannii* isolates tested with hydrochloric acid (HCL) and Acetic acid (AA). Optical density on the y axis refers to the average biofilm biomass for all isolates tested at the range of acid dilutions tested. The error bars represent the standard error.
(TIF)
