# Supplementary figures and images for: The Antibacterial Activity of Acetic Acid against Biofilm-Producing Pathogens of Relevance to Burns Patients
Source: PLoS One. 2015 Sep 9;10(9):e0136190. doi: 10.1371/journal.pone.0136190 (PMC4566994; doi:10.1371/journal.pone.0136190)

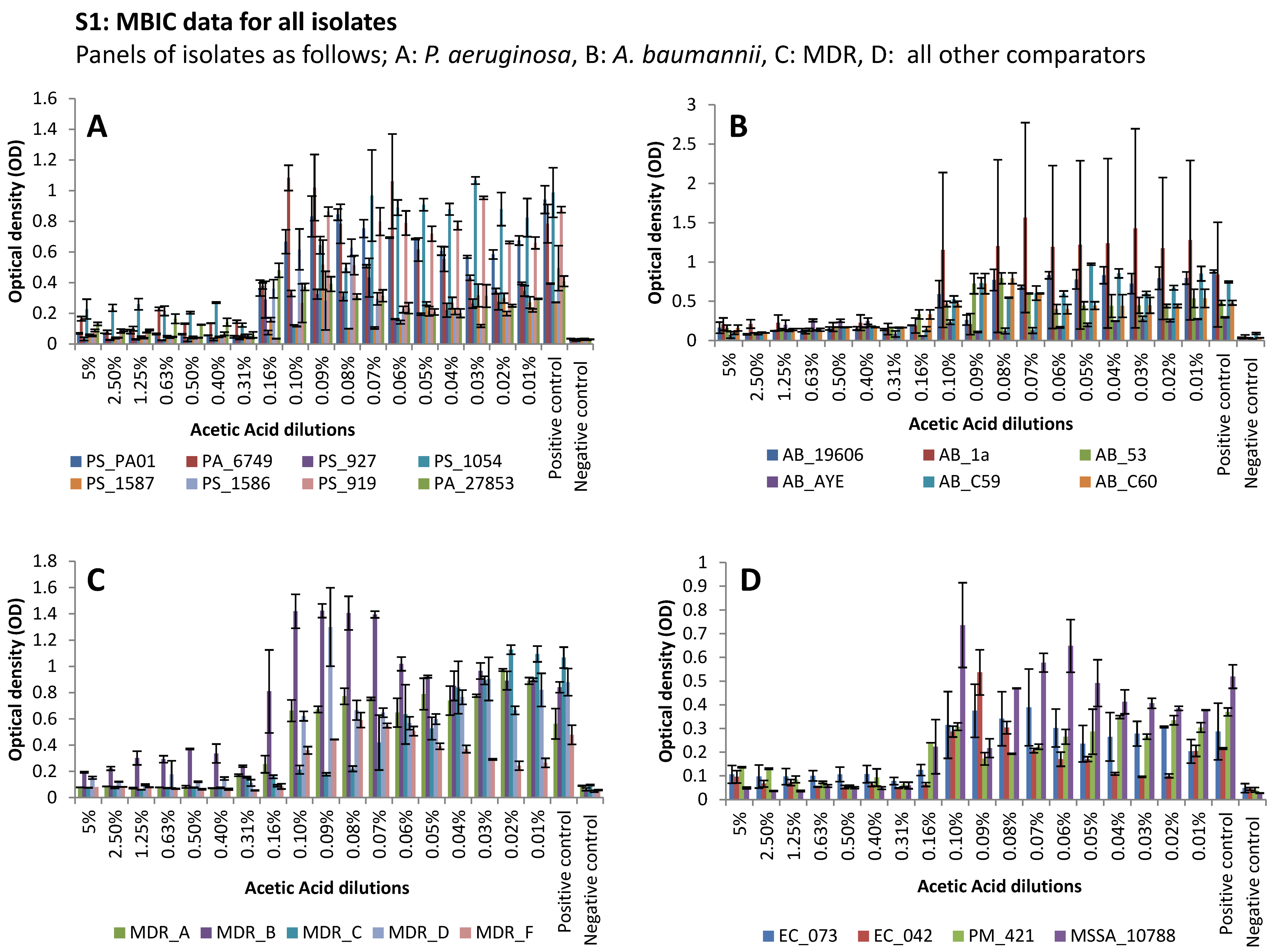

Supplement: S1 Fig — Panels of isolates as follows; A: P. aeruginosa, B: A. baumannii, C: MDR, D: all other comparators (TIF) [file pone.0136190.s001.tif]

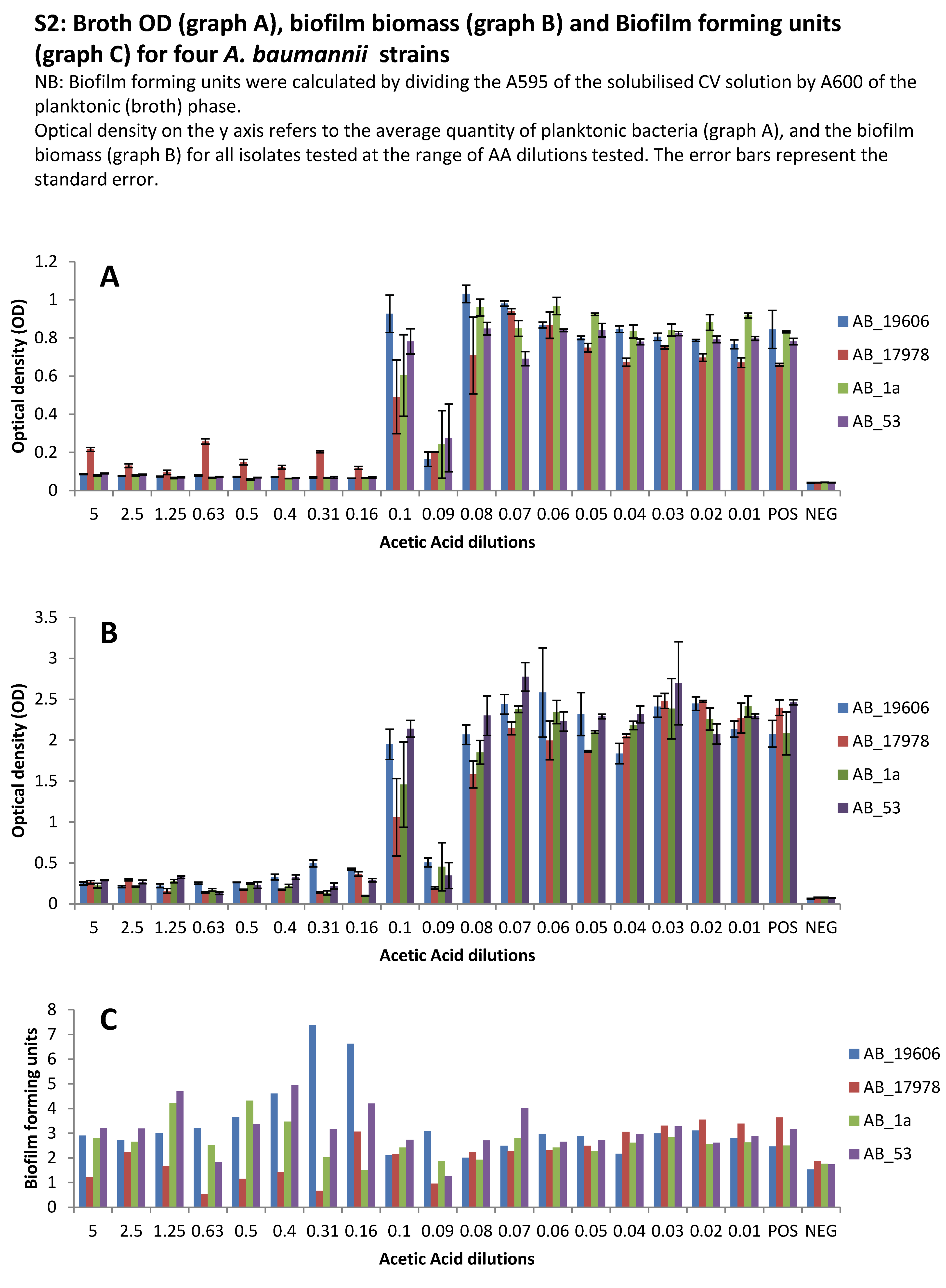

Supplement: S2 Fig — NB: Biofilm forming units were calculated by dividing the A595 of the solubilised CV solution by A600 of the planktonic (broth) phase. Optical density on the y axis refers to the average quantity of planktonic bacteria (graph A), and the biofilm biomass (graph B) for all isolates tested at the range of AA dilutions tested. The error bars represent the standard error. (TIF) [file pone.0136190.s002.tif]

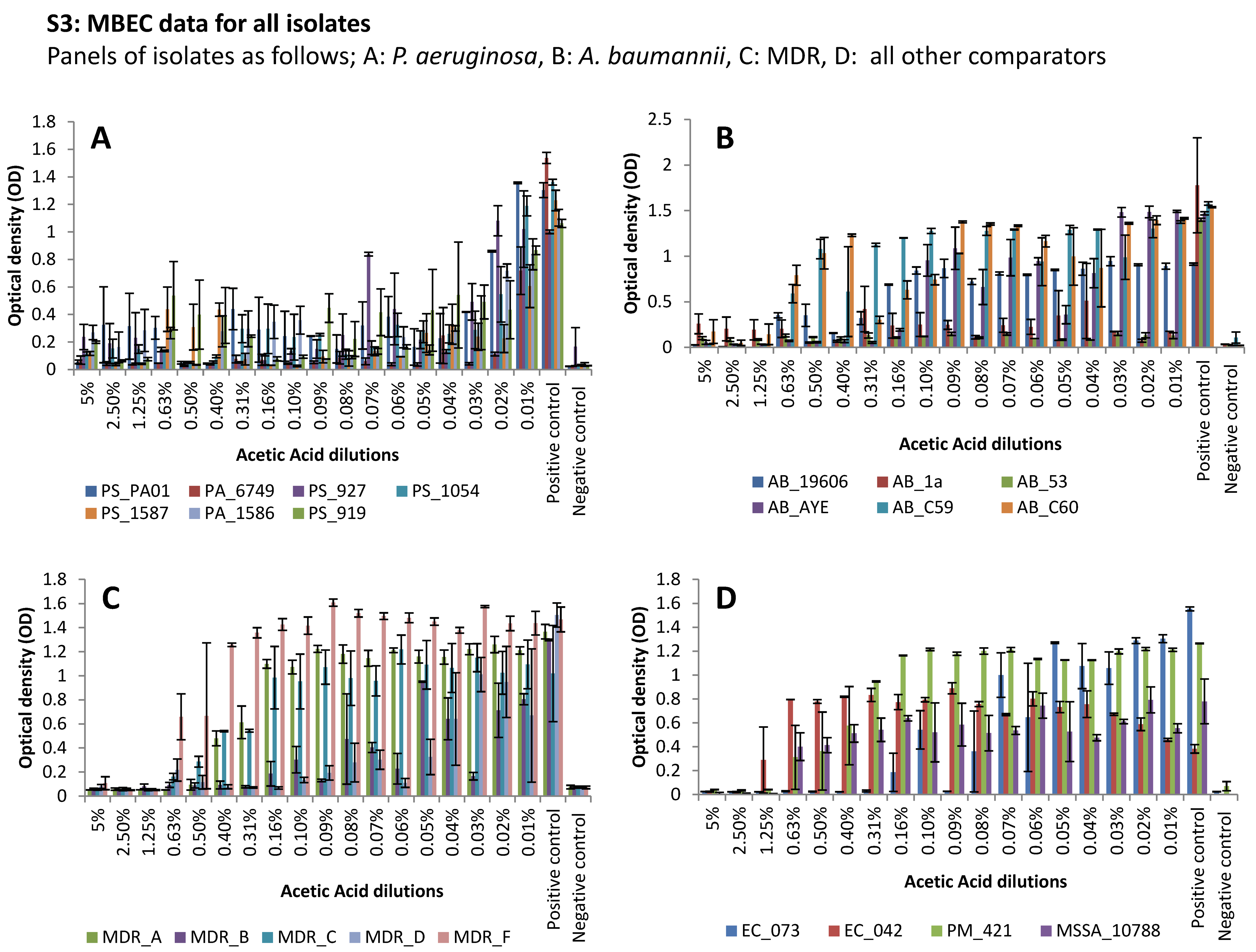

Supplement: S3 Fig — Panels of isolates as follows; A: P. aeruginosa, B: A. baumannii, C: MDR, D: all other comparators. (TIF) [file pone.0136190.s003.tif]

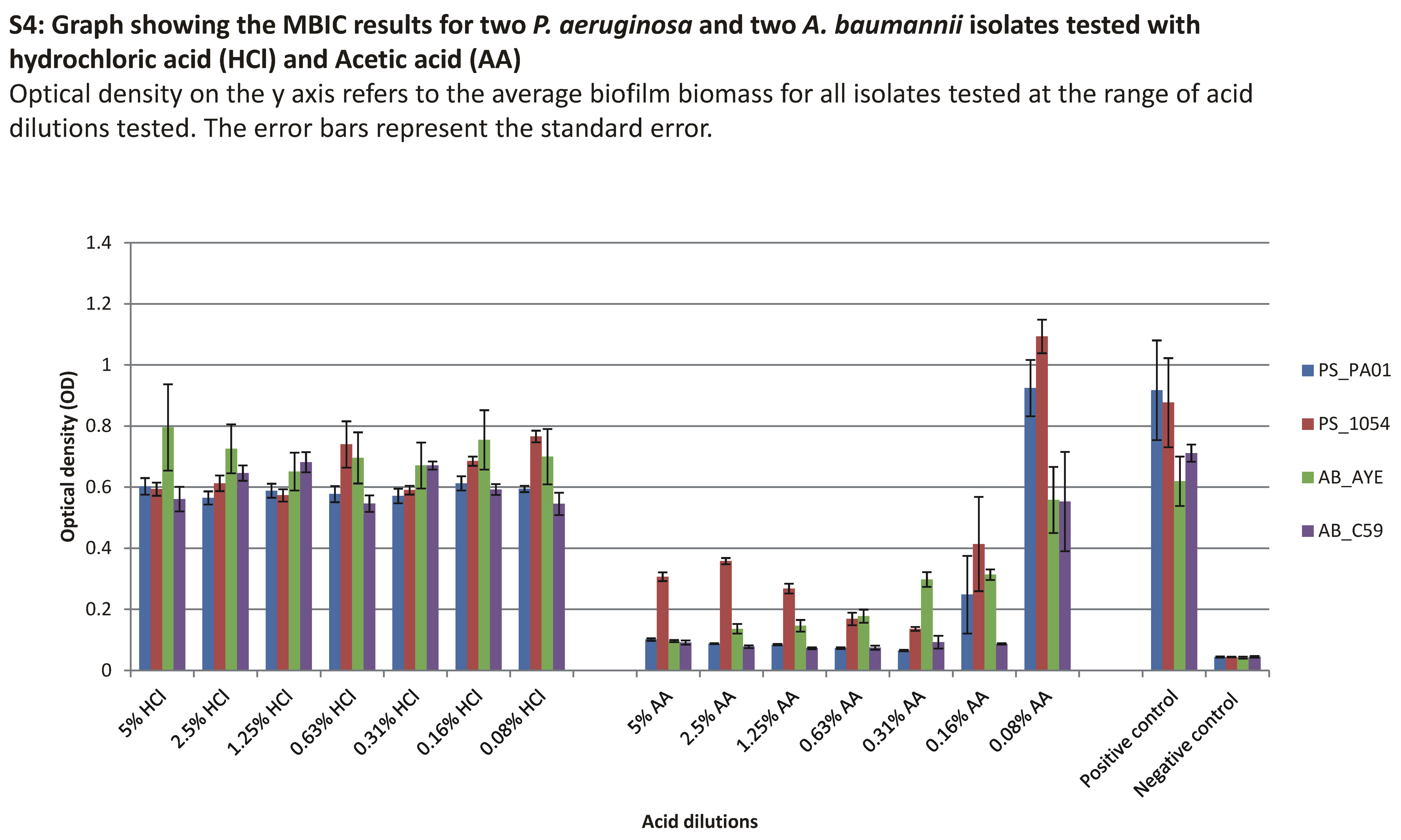

Supplement: S4 Fig — Optical density on the y axis refers to the average biofilm biomass for all isolates tested at the range of acid dilutions tested. The error bars represent the standard error. (TIF) [file pone.0136190.s004.tif]
